# Crystal structure of (5-chloro-2-hy­droxy­phen­yl)(3-methyl­isoxazolo[5,4-*b*]pyridin-5-yl)methanone

**DOI:** 10.1107/S2056989015019635

**Published:** 2015-10-24

**Authors:** Rajamani Raja, Nataraj Poomathi, Paramasivam T. Perumal, A. SubbiahPandi

**Affiliations:** a Department of Physics, Presidency College (Autonomous), Chennai 600 005, India; b Organic Chemistry Division, CSIR Central Leather Research Institute, Adyar, Chennai 600 020, India

**Keywords:** crystal structure, polyfunctional pyridines, isoxazole, O—H⋯O hydrogen bonds, C—H⋯N hydrogen bonds

## Abstract

In the title compound, C_14_H_9_ClN_2_O_3_, the fused pyridine and isoxazole rings are approximately planar, making a dihedral angle of 1.14 (16)°. The mol­ecule is twisted with the benzene ring and the mean plane through the fused pyridine-isoxazole ring system being inclined to one another by 47.03 (13)°. There is an intra­molecular O—H⋯O hydrogen bond forming an *S*(6) ring motif. In the crystal, mol­ecules are linked by C—H⋯N hydrogen bonds, forming chains propagating along [001]. The chains are linked by slipped parallel π–π inter­actions, involving inversion-related benzene rings, forming slabs lying parallel to the *bc* plane {inter-centroid distance = 3.770 (2) Å].

## Related literature   

For various applications of polyfunctional pyridines, see: Knyazhanskii *et al.* (1996 ▸); Kürfurst *et al.* (1989 ▸); Enyedy *et al.* (2003 ▸); Arora & Knaus (1999 ▸); Kim *et al.* 2004 ▸); Pillai *et al.*(2003 ▸).
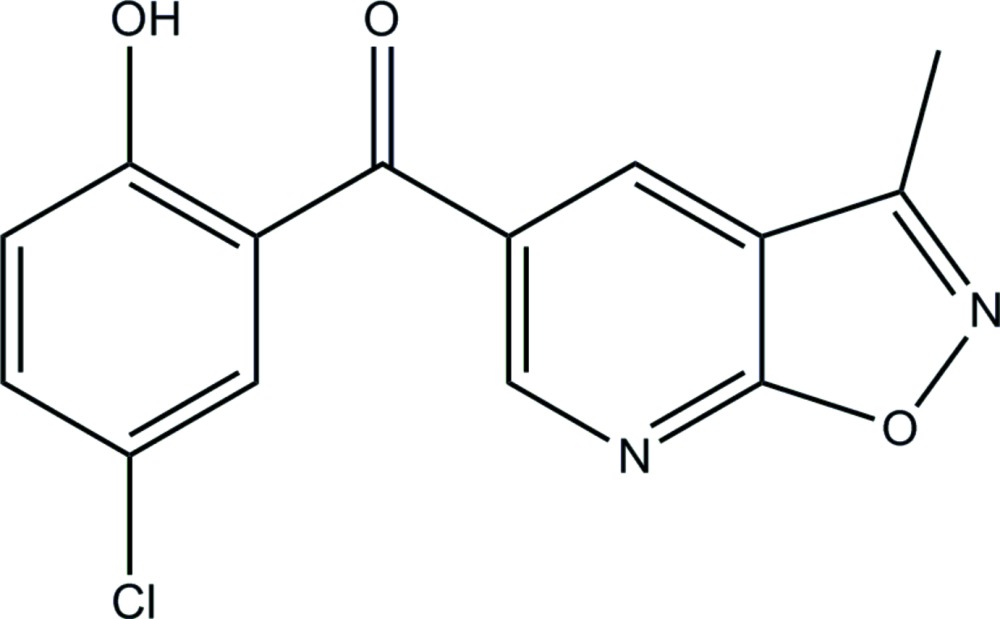


## Experimental   

### Crystal data  

C_14_H_9_ClN_2_O_3_
*M* *_r_* = 288.68
Monoclinic, 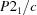

*a* = 11.0317 (10) Å
*b* = 11.8701 (10) Å
*c* = 11.1220 (9) Å
β = 118.675 (2)°
*V* = 1277.78 (19) Å^3^
*Z* = 4
Mo *K*α radiation
μ = 0.31 mm^−1^
*T* = 293 K
0.35 × 0.30 × 0.25 mm

### Data collection  

Bruker SMART APEXII CCD diffractometer
Absorption correction: multi-scan (*SADABS*; Bruker, 2008 ▸) *T*_min_ = 0.900, *T*_max_ = 0.927
17705 measured reflections
2250 independent reflections
1763 reflections with *I* > 2σ(*I*)
*R*_int_ = 0.022

### Refinement  

*R*[*F* ^2^ > 2σ(*F* ^2^)] = 0.048
*wR*(*F* ^2^) = 0.110
*S* = 1.13
2250 reflections
182 parameters
H-atom parameters constrained
Δρ_max_ = 0.28 e Å^−3^
Δρ_min_ = −0.42 e Å^−3^

### 

Data collection: *APEX2* (Bruker, 2008 ▸); cell refinement: *SAINT* (Bruker, 2008 ▸); data reduction: *SAINT*; program(s) used to solve structure: *SHELXS97* (Sheldrick, 2008 ▸); program(s) used to refine structure: *SHELXL97* (Sheldrick, 2008 ▸); molecular graphics: *ORTEP-3 for Windows* (Farrugia, 2012 ▸); software used to prepare material for publication: *SHELXL97* and *PLATON* (Spek, 2009 ▸).

## Supplementary Material

Crystal structure: contains datablock(s) global, I. DOI: 10.1107/S2056989015019635/su5220sup1.cif

Structure factors: contains datablock(s) I. DOI: 10.1107/S2056989015019635/su5220Isup2.hkl

Supporting information file. DOI: 10.1107/S2056989015019635/su5220Isup3.cml

**Fig. 1.**
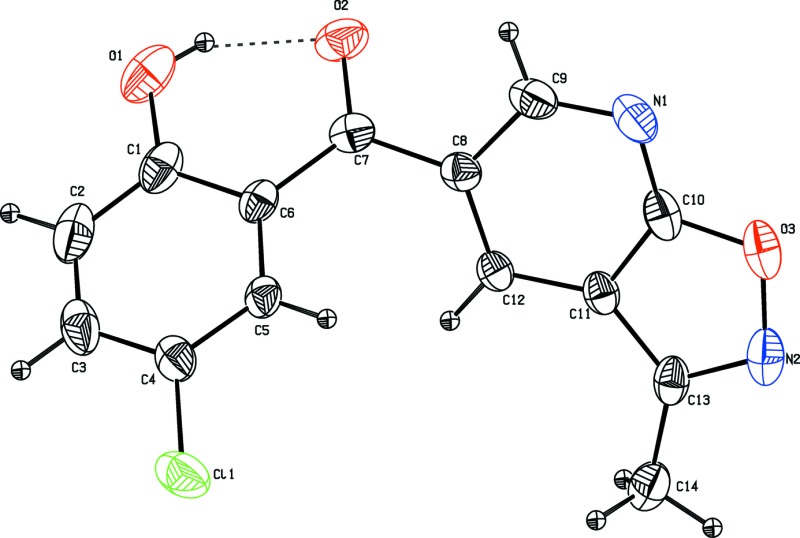
The molecular structure of the title compound, with atom labelling. Displacement ellipsoids are drawn at the 30% probability level.

**Fig. 2.**
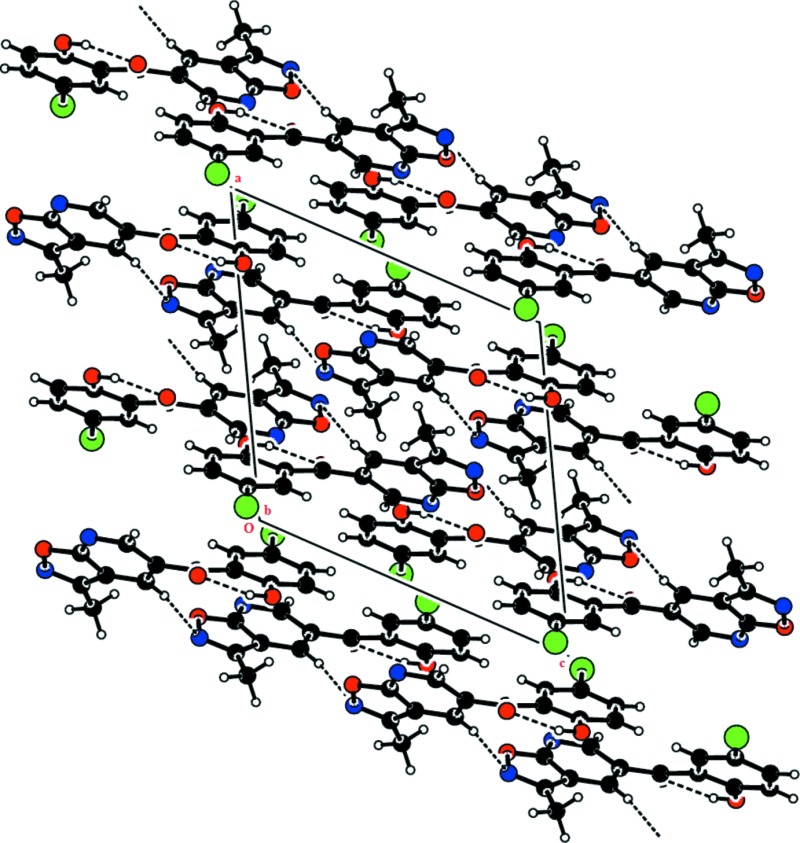
A view along the b-axis of the crystal packing of the title compound. The hydrogen bonds are shown as dashed lines (see Table 1 for details).

. DOI: 10.1107/S2056989015019635/su5220fig1.tif
The mol­ecular structure of the title compound, with atom labelling. Displacement ellipsoids are drawn at the 30% probability level.

b- . DOI: 10.1107/S2056989015019635/su5220fig2.tif
A view along the *b-*axis of the crystal packing of the title compound. The hydrogen bonds are shown as dashed lines (see Table 1 for details).

CCDC reference: 1431889

Additional supporting information:  crystallographic information; 3D view; checkCIF report

## Figures and Tables

**Table 1:** Hydrogen-bond geometry (, )

*D*H*A*	*D*H	H*A*	*D* *A*	*D*H*A*
O1H1O2	0.82	1.84	2.561(4)	145
C12H12N2^i^	0.93	2.40	3.315(4)	168

Symmetry code: (i) 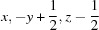.

## supplementary crystallographic information

### S1. Chemical context

Poly-functional pyridines are an inter­esting class of compounds due to their optical properties (Knyazhanskii *et al.*, 1996; Kürfurst *et al.*, 1989), and their biological activities (Enyedy *et al.*, 2003), such as anti­convulsants (Arora *et al.*, 1999), anti­histaminic reagents (Kim *et al.*, 2004), and cardivascular disorder treatments (Pillai *et al.*, 2003). In view of such facts we herein report on the synthesis and crystal structure of the new title poly-functional pyridine compound.

### S2. Structural commentary

In the title compound, Fig. 1, the fused pyridine ring (N1/C8—C12) and isoxazole ring (O3/N2/C13/C11/C10) are almost coplanar being inclined to one another by 1.14 (16) °. The molecule is twisted with the benzene ring (C1—C6) and the mean plane through the fused pyridine-isoxazole ring system being inclined to one another by 47.03 (13) °. The molecular conformation is partly determined by the intra­molecular O—H···O hydrogen bond which forms an S(6) ring motif.

In the crystal, molecules are linked by C—H···N hydrogen bond to form chains propagating along the *c* axis direction (Table 1 and Fig. 2). The chains are linked by slipped parallel π—π inter­actions, involving inversion related 5-chloro-2-hy­droxy­phenyl rings, forming slabs parallel to the *bc*-plane; see Fig. 2 [*Cg*3—*Cg*3^i^ = 3.770 (2) Å, inter-planar distance = 3.4094 (14) Å, slippage = 1.609 Å; *Cg*3 is the centroid of ring (C1—C6); symmetry code: (i) −*x*, −*y*, 2 − *z*].

### S3. Synthesis and crystallization

To a mixture of 6-chloro-3-formyl­chromone (1 mmol) and 3-methyl­isoxazol-5-amine (1 mmol) in ethanol (3 ml) was added a catalytic amount (0.050 mmol) of In(OTf)_3_ and the mixture was refluxed for about 20 min. The precipitated solid was filtered and dried under vacuum to afford the pure product in 87% yield. The purified compound was recrystallized from ethanol and DMSO-D_6_ by slow evaporation giving colourless block-like crystals.

### S4. Refinement

Crystal data, data collection and structure refinement details are summarized in Table 2. The OH and C-bound H atoms were positioned geometrically (O—H = 0.82 Å, C–H = 0.93–0.96 Å) and allowed to ride on their parent atoms, with *U*_iso_(H) = 1.5*U*_eq_(O,*C*) for hydroxyl and methyl H atoms and 1.2*U*_eq_(C) for other H atoms.

### Crystal data

**Table:**

C_14_H_9_ClN_2_O_3_	*F*(000) = 592
*M**_r_* = 288.68	*D*_x_ = 1.501 Mg m^−^^3^
Monoclinic, *P*2_1_/*c*	Mo *K*α radiation, λ = 0.71073 Å
Hall symbol: -P 2ybc	Cell parameters from 1763 reflections
*a* = 11.0317 (10) Å	θ = 2.1–25.0°
*b* = 11.8701 (10) Å	µ = 0.31 mm^−^^1^
*c* = 11.1220 (9) Å	*T* = 293 K
β = 118.675 (2)°	Block, colourless
*V* = 1277.78 (19) Å^3^	0.35 × 0.30 × 0.25 mm
*Z* = 4	

### Data collection

**Table:**

Bruker SMART APEXII CCD diffractometer	2250 independent reflections
Radiation source: fine-focus sealed tube	1763 reflections with *I* > 2σ(*I*)
Graphite monochromator	*R*_int_ = 0.022
ω and φ scans	θ_max_ = 25.0°, θ_min_ = 2.1°
Absorption correction: multi-scan (*SADABS*; Bruker, 2008)	*h* = −13→13
*T*_min_ = 0.900, *T*_max_ = 0.927	*k* = −14→14
17705 measured reflections	*l* = −13→13

### Refinement

**Table:**

Refinement on *F*^2^	Primary atom site location: structure-invariant direct methods
Least-squares matrix: full	Secondary atom site location: difference Fourier map
*R*[*F*^2^ > 2σ(*F*^2^)] = 0.048	Hydrogen site location: inferred from neighbouring sites
*wR*(*F*^2^) = 0.110	H-atom parameters constrained
*S* = 1.13	*w* = 1/[σ^2^(*F*_o_^2^) + (0.0188*P*)^2^ + 1.3834*P*] where *P* = (*F*_o_^2^ + 2*F*_c_^2^)/3
2250 reflections	(Δ/σ)_max_ < 0.001
182 parameters	Δρ_max_ = 0.28 e Å^−^^3^
0 restraints	Δρ_min_ = −0.42 e Å^−^^3^

### Special details

**Table:**

Geometry. All e.s.d.'s (except the e.s.d. in the dihedral angle between two l.s. planes) are estimated using the full covariance matrix. The cell e.s.d.'s are taken into account individually in the estimation of e.s.d.'s in distances, angles and torsion angles; correlations between e.s.d.'s in cell parameters are only used when they are defined by crystal symmetry. An approximate (isotropic) treatment of cell e.s.d.'s is used for estimating e.s.d.'s involving l.s. planes.
Refinement. Refinement of *F*^2^ against ALL reflections. The weighted *R*-factor *wR* and goodness of fit *S* are based on *F*^2^, conventional *R*-factors *R* are based on *F*, with *F* set to zero for negative *F*^2^. The threshold expression of *F*^2^ > σ(*F*^2^) is used only for calculating *R*-factors(gt) *etc*. and is not relevant to the choice of reflections for refinement. *R*-factors based on *F*^2^ are statistically about twice as large as those based on *F*, and *R*- factors based on ALL data will be even larger.

### Fractional atomic coordinates and isotropic or equivalent isotropic displacement parameters (Å^2^)

**Table:**

	*x*	*y*	*z*	*U*_iso_*/*U*_eq_	
C1	0.1833 (3)	−0.0368 (3)	0.9816 (3)	0.0592 (8)	
C2	0.1378 (3)	0.0264 (4)	0.8629 (3)	0.0738 (10)	
H2	0.1386	−0.0051	0.7868	0.089*	
C3	0.0917 (3)	0.1343 (3)	0.8560 (3)	0.0711 (10)	
H3	0.0614	0.1755	0.7755	0.085*	
C4	0.0900 (3)	0.1827 (3)	0.9696 (3)	0.0541 (7)	
C5	0.1386 (2)	0.1234 (2)	1.0890 (2)	0.0432 (6)	
H5	0.1384	0.1564	1.1648	0.052*	
C6	0.1887 (2)	0.0133 (2)	1.0988 (3)	0.0436 (6)	
C7	0.2418 (2)	−0.0531 (2)	1.2263 (3)	0.0455 (6)	
C8	0.2796 (2)	−0.0001 (2)	1.3606 (3)	0.0424 (6)	
C9	0.2544 (3)	−0.0637 (2)	1.4535 (3)	0.0578 (7)	
H9	0.2125	−0.1336	1.4243	0.069*	
C10	0.3469 (3)	0.0675 (3)	1.6116 (3)	0.0528 (7)	
C11	0.3811 (2)	0.1373 (2)	1.5332 (2)	0.0414 (6)	
C12	0.3455 (2)	0.1025 (2)	1.4011 (2)	0.0388 (6)	
H12	0.3652	0.1460	1.3430	0.047*	
C13	0.4447 (3)	0.2330 (2)	1.6185 (2)	0.0471 (6)	
C14	0.5010 (3)	0.3350 (3)	1.5876 (3)	0.0607 (8)	
H14A	0.5386	0.3840	1.6659	0.091*	
H14B	0.4286	0.3733	1.5107	0.091*	
H14C	0.5726	0.3140	1.5662	0.091*	
N1	0.2861 (3)	−0.0312 (2)	1.5798 (3)	0.0656 (7)	
N2	0.4480 (3)	0.2213 (2)	1.7365 (2)	0.0640 (7)	
O1	0.2205 (3)	−0.1444 (2)	0.9793 (3)	0.0850 (8)	
H1	0.2460	−0.1732	1.0547	0.127*	
O2	0.2567 (2)	−0.15626 (16)	1.2243 (2)	0.0661 (6)	
O3	0.3854 (2)	0.1149 (2)	1.73518 (19)	0.0709 (6)	
Cl1	0.02472 (9)	0.31717 (7)	0.95930 (9)	0.0802 (3)	

### Atomic displacement parameters (Å^2^)

**Table:**

	*U*^11^	*U*^22^	*U*^33^	*U*^12^	*U*^13^	*U*^23^
C1	0.0495 (17)	0.074 (2)	0.0569 (18)	−0.0167 (15)	0.0281 (14)	−0.0221 (16)
C2	0.071 (2)	0.106 (3)	0.0519 (19)	−0.033 (2)	0.0359 (17)	−0.0268 (19)
C3	0.061 (2)	0.102 (3)	0.0392 (16)	−0.0353 (19)	0.0150 (14)	0.0022 (17)
C4	0.0383 (14)	0.0663 (18)	0.0424 (15)	−0.0144 (13)	0.0070 (12)	0.0058 (13)
C5	0.0346 (13)	0.0518 (15)	0.0355 (13)	−0.0093 (11)	0.0106 (11)	−0.0045 (11)
C6	0.0340 (13)	0.0525 (15)	0.0442 (14)	−0.0113 (11)	0.0188 (11)	−0.0120 (12)
C7	0.0335 (13)	0.0425 (15)	0.0556 (16)	0.0001 (11)	0.0175 (12)	−0.0020 (12)
C8	0.0371 (13)	0.0408 (14)	0.0444 (14)	0.0072 (11)	0.0156 (11)	0.0055 (11)
C9	0.0558 (17)	0.0484 (16)	0.0618 (18)	0.0011 (13)	0.0222 (15)	0.0138 (14)
C10	0.0456 (16)	0.073 (2)	0.0380 (14)	0.0126 (14)	0.0183 (12)	0.0119 (14)
C11	0.0364 (13)	0.0506 (15)	0.0355 (13)	0.0095 (11)	0.0159 (11)	0.0066 (11)
C12	0.0336 (12)	0.0447 (14)	0.0365 (12)	0.0059 (11)	0.0154 (10)	0.0061 (11)
C13	0.0381 (14)	0.0623 (17)	0.0339 (13)	0.0125 (12)	0.0116 (11)	−0.0022 (12)
C14	0.0591 (18)	0.0647 (19)	0.0491 (16)	−0.0026 (15)	0.0185 (14)	−0.0124 (14)
N1	0.0707 (17)	0.0709 (18)	0.0530 (15)	0.0019 (14)	0.0277 (13)	0.0197 (13)
N2	0.0630 (16)	0.088 (2)	0.0388 (13)	0.0124 (14)	0.0224 (12)	−0.0035 (13)
O1	0.0909 (17)	0.0841 (17)	0.0906 (17)	−0.0042 (14)	0.0521 (15)	−0.0375 (14)
O2	0.0642 (13)	0.0443 (12)	0.0777 (15)	0.0077 (10)	0.0243 (11)	−0.0065 (10)
O3	0.0781 (15)	0.0984 (18)	0.0404 (11)	0.0073 (13)	0.0317 (11)	0.0092 (11)
Cl1	0.0684 (5)	0.0677 (5)	0.0732 (6)	−0.0036 (4)	0.0090 (4)	0.0258 (4)

### Geometric parameters (Å, º)

**Table:**

C1—O1	1.345 (4)	C9—N1	1.331 (4)
C1—C2	1.386 (5)	C9—H9	0.9300
C1—C6	1.408 (4)	C10—N1	1.312 (4)
C2—C3	1.366 (5)	C10—O3	1.351 (3)
C2—H2	0.9300	C10—C11	1.380 (4)
C3—C4	1.396 (4)	C11—C12	1.390 (3)
C3—H3	0.9300	C11—C13	1.430 (4)
C4—C5	1.365 (4)	C12—H12	0.9300
C4—Cl1	1.733 (3)	C13—N2	1.302 (3)
C5—C6	1.403 (4)	C13—C14	1.474 (4)
C5—H5	0.9300	C14—H14A	0.9600
C6—C7	1.476 (4)	C14—H14B	0.9600
C7—O2	1.237 (3)	C14—H14C	0.9600
C7—C8	1.485 (4)	N2—O3	1.436 (3)
C8—C12	1.378 (3)	O1—H1	0.8200
C8—C9	1.411 (4)		
			
O1—C1—C2	118.0 (3)	N1—C9—H9	117.5
O1—C1—C6	122.8 (3)	C8—C9—H9	117.5
C2—C1—C6	119.2 (3)	N1—C10—O3	121.1 (3)
C3—C2—C1	121.1 (3)	N1—C10—C11	128.7 (3)
C3—C2—H2	119.5	O3—C10—C11	110.2 (3)
C1—C2—H2	119.5	C10—C11—C12	117.7 (3)
C2—C3—C4	120.1 (3)	C10—C11—C13	104.7 (2)
C2—C3—H3	120.0	C12—C11—C13	137.5 (2)
C4—C3—H3	120.0	C8—C12—C11	116.4 (2)
C5—C4—C3	120.0 (3)	C8—C12—H12	121.8
C5—C4—Cl1	119.7 (2)	C11—C12—H12	121.8
C3—C4—Cl1	120.4 (2)	N2—C13—C11	110.5 (3)
C4—C5—C6	120.8 (3)	N2—C13—C14	120.7 (3)
C4—C5—H5	119.6	C11—C13—C14	128.7 (2)
C6—C5—H5	119.6	C13—C14—H14A	109.5
C5—C6—C1	118.8 (3)	C13—C14—H14B	109.5
C5—C6—C7	122.2 (2)	H14A—C14—H14B	109.5
C1—C6—C7	119.0 (3)	C13—C14—H14C	109.5
O2—C7—C6	120.4 (2)	H14A—C14—H14C	109.5
O2—C7—C8	117.6 (2)	H14B—C14—H14C	109.5
C6—C7—C8	122.0 (2)	C10—N1—C9	112.6 (2)
C12—C8—C9	119.5 (2)	C13—N2—O3	107.7 (2)
C12—C8—C7	123.5 (2)	C1—O1—H1	109.5
C9—C8—C7	116.8 (2)	C10—O3—N2	106.9 (2)
N1—C9—C8	125.0 (3)		
			
O1—C1—C2—C3	176.6 (3)	C7—C8—C9—N1	177.5 (3)
C6—C1—C2—C3	−3.4 (5)	N1—C10—C11—C12	1.4 (4)
C1—C2—C3—C4	0.1 (5)	O3—C10—C11—C12	−178.4 (2)
C2—C3—C4—C5	2.1 (4)	N1—C10—C11—C13	179.9 (3)
C2—C3—C4—Cl1	−177.2 (2)	O3—C10—C11—C13	0.1 (3)
C3—C4—C5—C6	−0.9 (4)	C9—C8—C12—C11	−1.1 (3)
Cl1—C4—C5—C6	178.34 (18)	C7—C8—C12—C11	−176.2 (2)
C4—C5—C6—C1	−2.4 (4)	C10—C11—C12—C8	−0.4 (3)
C4—C5—C6—C7	−180.0 (2)	C13—C11—C12—C8	−178.3 (3)
O1—C1—C6—C5	−175.5 (2)	C10—C11—C13—N2	0.0 (3)
C2—C1—C6—C5	4.4 (4)	C12—C11—C13—N2	178.0 (3)
O1—C1—C6—C7	2.2 (4)	C10—C11—C13—C14	−179.6 (3)
C2—C1—C6—C7	−177.9 (3)	C12—C11—C13—C14	−1.6 (5)
C5—C6—C7—O2	164.1 (2)	O3—C10—N1—C9	179.3 (3)
C1—C6—C7—O2	−13.5 (4)	C11—C10—N1—C9	−0.6 (4)
C5—C6—C7—C8	−16.1 (4)	C8—C9—N1—C10	−1.2 (4)
C1—C6—C7—C8	166.3 (2)	C11—C13—N2—O3	0.0 (3)
O2—C7—C8—C12	140.6 (3)	C14—C13—N2—O3	179.6 (2)
C6—C7—C8—C12	−39.3 (4)	N1—C10—O3—N2	−179.9 (2)
O2—C7—C8—C9	−34.7 (3)	C11—C10—O3—N2	−0.1 (3)
C6—C7—C8—C9	145.5 (2)	C13—N2—O3—C10	0.0 (3)
C12—C8—C9—N1	2.1 (4)		

### Hydrogen-bond geometry (Å, º)

**Table:**

*D*—H···*A*	*D*—H	H···*A*	*D*···*A*	*D*—H···*A*
O1—H1···O2	0.82	1.84	2.561 (4)	145
C12—H12···N2^i^	0.93	2.40	3.315 (4)	168

Symmetry code: (i) *x*, −*y*+1/2, *z*−1/2.
