# Supplementary figures and images for: Crystal structure of (5-chloro-2-hy­droxy­phen­yl)(3-methyl­isoxazolo[5,4-b]pyridin-5-yl)methanone
Source: Acta Crystallogr E Crystallogr Commun. 2015 Oct 24;71(Pt 11):o875–6. doi: 10.1107/S2056989015019635 (PMC4645069; doi:10.1107/S2056989015019635)

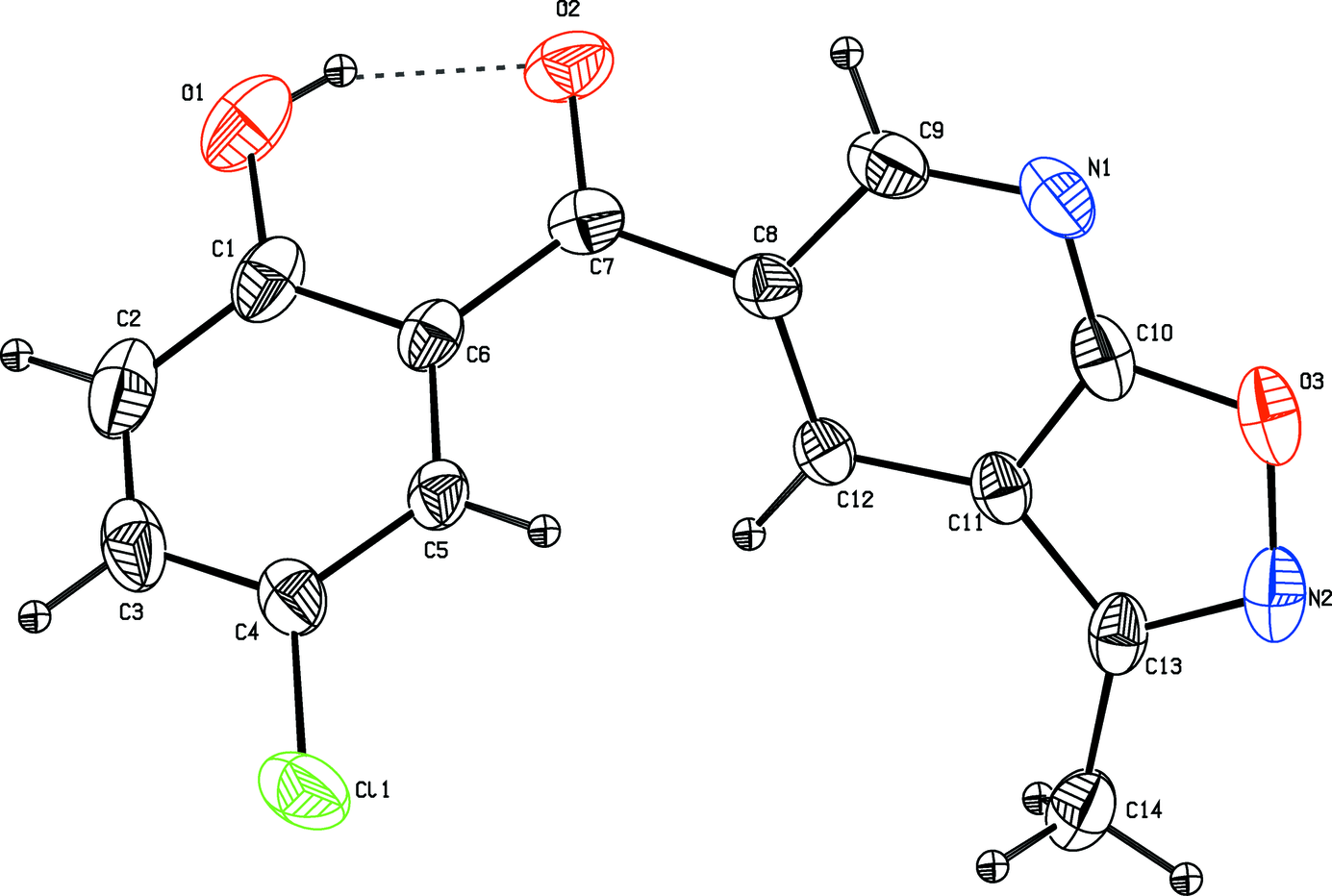

Supplement: Supplementary file 4 [file e-71-0o875-fig1.tif]

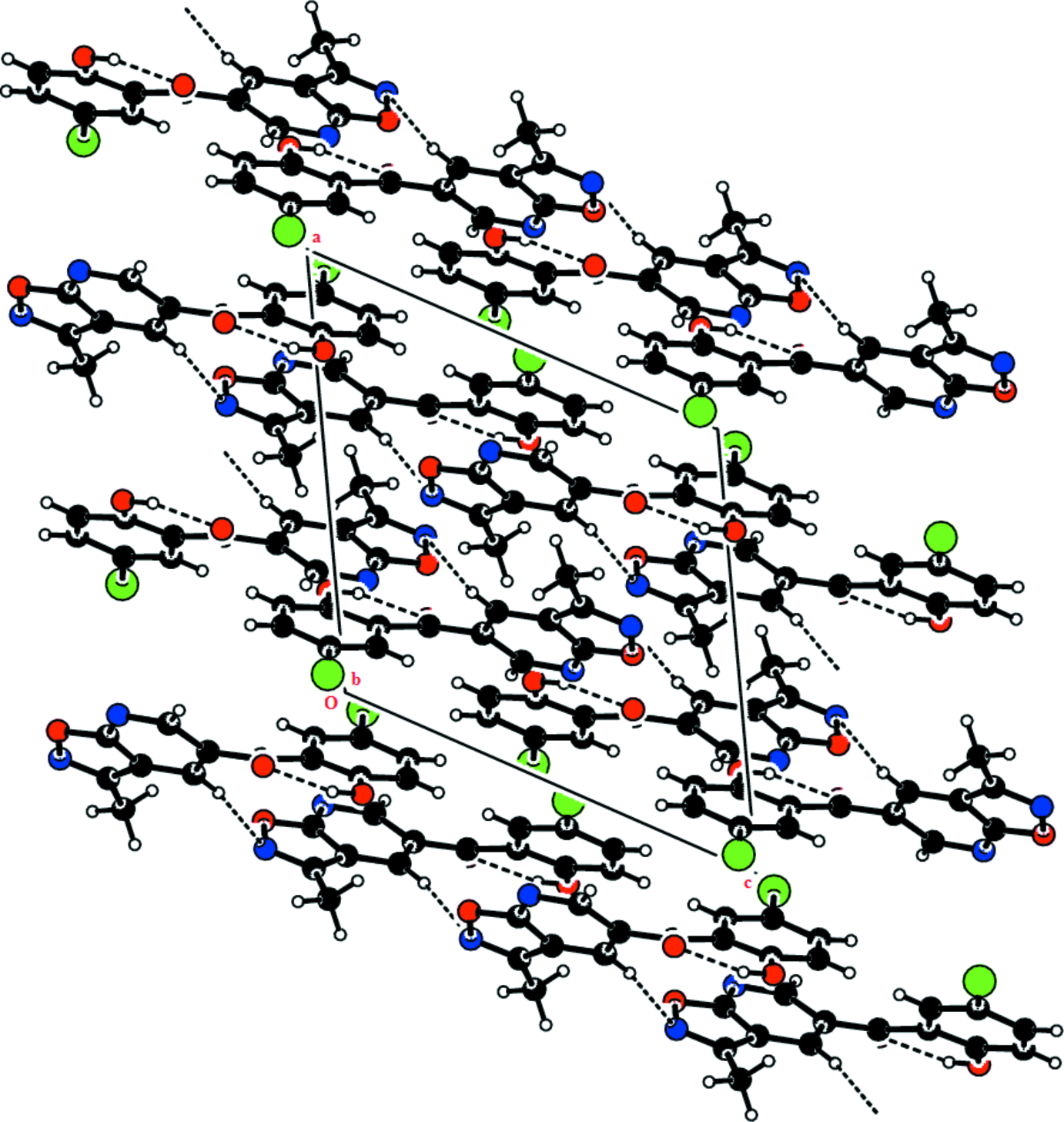

Supplement: Supplementary file 5 [file e-71-0o875-fig2.tif]
